# Inflammatory Neuropathy Consortium base (INCbase): a protocol of a global prospective observational cohort study for the development of a prediction model for treatment response in chronic inflammatory demyelinating polyneuropathy

**DOI:** 10.1186/s12883-024-03903-w

**Published:** 2024-10-25

**Authors:** Milou R. Michael, Luuk Wieske, Jeffrey A. Allen, Michael P. Lunn, Kathrin Doppler, Cheng-Yin Tan, Haruki Koike, Lars K. Markvardsen, Mahima Kapoor, Sung-Tsang Hsieh, Eduardo Nobile-Orazio, Bart C. Jacobs, Yusuf A. Rajabally, Ivana Basta, Paolo Ripellino, Luis Querol, Filip Eftimov, on behalf of the INCbase Consortium, Jeffrey A. Allen, Michael P. Lunn, Kathrin Doppler, Cheng-Yin Tan, Haruki Koike, Lars K. Markvardsen, Mahima Kapoor, Sung-Tsang Hsieh, Bart C. Jacobs, Ivana Basta, Paolo Ripellino, Filip Eftimov, Luis Querol, Gerardo Gutiérrez-Gutiérrez, Ivonne Jericó Pascual, Teresa Sevilla, German Moris, Eugenia Martinez-Hernandez, Arnau Llaurado-Gayete, Marie Theaudin, Andrea Humm, Thomas Hundsberger, Sara Nagy, Agustina Lascano, Jia-Ying Sung, Long-Sun Ro, Kuan-Lin Lai, Ahmet Hoke, Mamatha Pasnoor, Amro M. Stino, Karissa Gable, Michal Vytopil, Diana Castro

**Affiliations:** 1 Department of Neurology and Neurophysiology, Location AMC, Amsterdam Neuroscience, Amsterdam UMC, University of Amsterdam, Amsterdam, the Netherlands; 2 Department of Neurology, University of Minnesota, Minneapolis, USA; 3 Centre for Neuromuscular Disease, National Hospital for Neurology and Neurosurgery, Queen Square, London, UK; 4 Department of Neurology, University Hospital Würzburg, Würzburg, Germany; 5 Division of Neurology, Department of Medicine, University of Malaya, Kuala Lumpur, 50603 Malaysia; 6 Division of Neurology, Department of Internal Medicine, Faculty of Medicine, Saga University, Saga, Japan; 7 Department of Neurology, Aarhus University Hospital, Aarhus, Denmark; 8 Neurology Department, Alfred Hospital, Melbourne, VIC Australia; 9 Department of Neurology, National Taiwan University Hospital, Taipei City, Taiwan; 10 Neuromuscular and Neuroimmunology Service, Department of Medical Biotechnology and Translational Medicine, IRCCS Humanitas Research Institute, University of Milan, Milan, Italy; 11 Department of Neurology and Immunology, Erasmus MC University Medical Center, Rotterdam, The Netherlands; 12 Neuromuscular Service, Neurology, Queen Elizabeth Hospital Birmingham, Birmingham, UK; 13 Neurology Clinic, Medical faculty, University Clinical Center of Serbia, University of Belgrade, Belgrade, Serbia; 14 Department of Neurology, Neurocenter of Southern Switzerland EOC, Lugano, CH Switzerland; 15 Faculty of Biomedical Sciences, Università della Svizzera Italiana, Lugano, CH Switzerland; 16 Department of Neurology, Hospital de la Santa Creu i Sant Pau, Barcelona, Spain; 17 Department of Neurology, Amsterdam UMC, location AMC, Meibergdreef 9, Amsterdam, 1105 AZ The Netherlands

**Keywords:** Chronic inflammatory demyelinating polyneuropathy, Diagnosis, Prognosis, Outcome, Clinimetrics, Treatment, Biomarkers, Prediction model

## Abstract

**Background:**

INCbase is an international, multicenter prospective observational study using a customizable web-based modular registry to study the clinical, biological and electrophysiological variation and boundaries of chronic inflammatory demyelinating polyneuropathy (CIDP). The primary objective of INCbase is to develop and validate a clinical prediction model for treatment response.

**Methods:**

All patients meeting clinical criteria for CIDP can be included in INCbase. Collected data include demographics, clinical history, diagnostics and various domains of clinical outcomes. Data is collected at a minimum of every 6 months for two years, and more frequently at the discretion of the investigational site to allow for assessment of unexpected changes in treatment response or clinical status. Participants can be enrolled in various sub-studies designed to capture data relevant to specific groups of interest. Data is entered directly into the web-based data entry system by local investigators and/or participants. Collection and local storage of biomaterial is optional. To develop a clinical prediction model for treatment response, newly diagnosed patients with active disease warranting start of first-line treatment will be included. The study population will be split into a development and validation cohort. Univariate and multivariate logistic regression analysis will be used to identify and combine predictors at start of treatment for treatment response at six months. Model performance will be assessed through discrimination and calibration in an external validation cohort. The externally validated prediction model will be made available to researchers and clinicians on the INCbase website.

**Discussion:**

With this study, we aim to create a clinically relevant and implementable prediction model for treatment response to first line treatments in CIDP. INCbase enrollment started in April 2021, with 29 centers across 8 countries and 303 patients participating to date. This collaborative effort between academia, patient advocacy organizations and pharmaceutical industry will deepen our understanding of how to diagnose and treat CIDP.

**Supplementary Information:**

The online version contains supplementary material available at 10.1186/s12883-024-03903-w.

## Background

Chronic inflammatory demyelinating polyradiculoneuropathy (CIDP) is a rare and heterogeneous immune-mediated neuropathy with wide variability in clinical phenotype, pathophysiology, treatment responsiveness, and prognosis. While the “typical” subtype is characterized by progressive or relapsing motor and sensory symptoms, “variant” subtypes with different clinical features have been recognized, including distal, motor, sensory and (multi)focal CIDP [1]. The extent to which these phenotypic variants also differ in pathophysiology, treatment response and prognosis is unknown [2, 3]. The heterogeneity and rarity of CIDP precludes a complete understanding of the clinical and pathobiological boundaries of CIDP and its variants with conventional local or regional data collection platforms.

Three key domains of CIDP that are in need of improved characterization are those of treatment, diagnosis and prognosis. The prediction of treatment response is a crucial aspect of disease management in CIDP. Treatment of CIDP before the onset of widespread or severe axonal damage is essential to prevent potentially irreversible disability. Efficacy of first line induction therapies, such as intravenous immunoglobulins (IVIg) and corticosteroids, is well established [4, 5]. Although some clinical variants fare better with IVIg [1, 6, 7] and others with corticosteroids, efficacy is largely comparable across these therapies. For smaller subgroups of patients, treatment response is insufficient or absent, requiring escalation of immunotherapy. Poor treatment response may be explained by misdiagnosis, inactive disease in combination with irreversible axonal damage, inappropriate treatment selection, and undertreatment. There are currently no known factors by which to predict treatment response. At present, treatment choice and timing is determined by pragmatic rather than evidence based approaches, and prescribing features such as dosage and duration are often driven by patient preference, physician discretion, and previous experience. By identifying objective clinical predictors for response to first line treatment, treatment strategies may be tailored to individual patient characteristics. This proactive approach may enable faster symptom relief and prevent disease progression and consequent disability, while minimizing treatment-related risks and costs.

To address these and other future issues, a large cohort of prospectively followed patients with CIDP is needed. For this purpose, INCbase was initiated: a global, collaborative effort collecting standardized prospective data and biomaterial. The primary objective of INCbase is to develop and validate a model capable of predicting treatment response in patients with CIDP at the start of treatment. Other substudies in INCbase are mainly focusing on improving diagnostic accuracy and development of biomarkers to predict and monitor treatment response and disease activity.

In this paper we will first provide an outline of the overall INCbase infrastructure, inclusion and exclusion criteria and collection of data, after which we will focus on the development and validation of a prediction model for treatment response in CIDP (primary objective).

## Methods overall study: design, data collection, governance and ethics

### Overall study design

INCbase is a global, multicenter observational study in which standardized prospective longitudinal data is collected using a modular web-based registry. Clinical data and optional biomaterials will be collected according to a pre-specified protocol. Patients can be enrolled in either a “core” or “extended” module. The core module captures a minimal set of core data, whereas the extended module is derived from the International CIDP Outcome Study (ICOS) [8] and includes more study visits, additional outcomes, and data relevant to patients treated with plasma exchange (PE) and subcutaneous immunoglobulins, as part of two pre-defined substudies within INCbase. Additional data may be collected at scheduled or unexpected time points where heightened disease activity is suspected (e.g., after treatment initiation or relapse). A supplementary home assessment module will be made available to predefined groups of patients, including but not limited to stable patients starting treatment withdrawal or tapering. During home assessments patients perform grip strength measurements and complete patient reported outcomes at scheduled intervals between study visits.

### Inclusion criteria overall study

All patients with written informed consent, conforming to one of the clinical definitions of CIDP as described in the 2021 EAN/PNS criteria [1] are eligible for inclusion in INCbase, regardless of whether they fulfill the electrophysiological or supportive criteria, and irrespective of the presence auto-antibodies to nodal or paranodal antigens (autoimmune nodopathies). The clinical definitions of CIDP includes typical CIDP and its variants [1]:

#### Typical CIDP

All the following:

- Progressive or relapsing, symmetric, proximal and distal muscle weakness of upper and lower limbs, and sensory involvement of at least two limbs.
- Developing over at least 8 weeks.
- Absent or reduced tendon reflexes in all limbs.

#### CIDP variants

One of the following, but otherwise as in typical CIDP (tendon reflexes may be normal in unaffected limbs):

- Distal CIDP: distal sensory loss and muscle weakness predominantly in lower limbs.
- Multifocal CIDP: sensory loss and muscle weakness in a multifocal pattern, usually asymmetric, upper limb predominant, in more than one limb.
- Focal CIDP: sensory loss and muscle weakness in only one limb.
- Motor CIDP: motor symptoms and signs without sensory involvement.
- Sensory CIDP: sensory symptoms and signs without motor involvement.

### Exclusion criteria overall study

1. The presence of any condition that at the discretion of the study investigator or study participant, impairs the participants ability to provide accurate study information in a timely and reliable manner;
2. Any alternative diagnosis to the patients neuropathic disorder (e.g.,. hereditary neuropathy, POEMS, anti-MAG neuropathy, MMN) diagnosed according to international or local guidelines at baseline.

### Study procedures overall study

#### Clinical, diagnostic and treatment data at baseline

At baseline, clinical data collected for all patients includes epidemiological data (e.g. age, gender, medical history), diagnostic data (results obtained during diagnostic work-up as part of routine clinical care, such as qualitative values of nerve conduction studies, imaging, CSF, and nerve biopsy and all excluded diagnoses and conditions associated with CIDP), disease history and clinical course, and, if appropriate, data concerning any previous treatment(s) and related adverse events. Qualitative aspects of nerve conduction studies recorded include tested nerves and the presence and certainty of demyelinating features in each nerve according to the normative values of the laboratory and 2021 EAN/PNS definitions for demyelination [1].

Clinical assessment performed at baseline includes the Inflammatory Neuropathy Cause and Treatment Disability Score (INCAT-DS) [9], the Medical Research Council sum score (MRC-SS) [10], the modified Inflammatory Neuropathy Cause and Treatment Sensory Sum Score (mISS) [11], tendon reflexes, ataxia, and grip strength. Questionnaires filled in by participants (patient reported outcome measures, PROMs) include the Inflammatory Rasch-built Overall Disability Scale (I-RODS) [12] and the EuroQol EQ-5D health questionnaire [13]. In the extended and home assessment module, additional questionnaires consist of the Rasch-built-7-item modified fatigue severity scale (Rasch-FSS) [14], the Pain Intensity Numerical Rating Scale (PI-NRS), a treatment satisfaction questionnaire, the Hospital Anxiety and Depression Scale (HADS) [15] and the General Self Efficacy Scale (GSES) [16].

#### Follow-up schedule

**Fig. 1:**
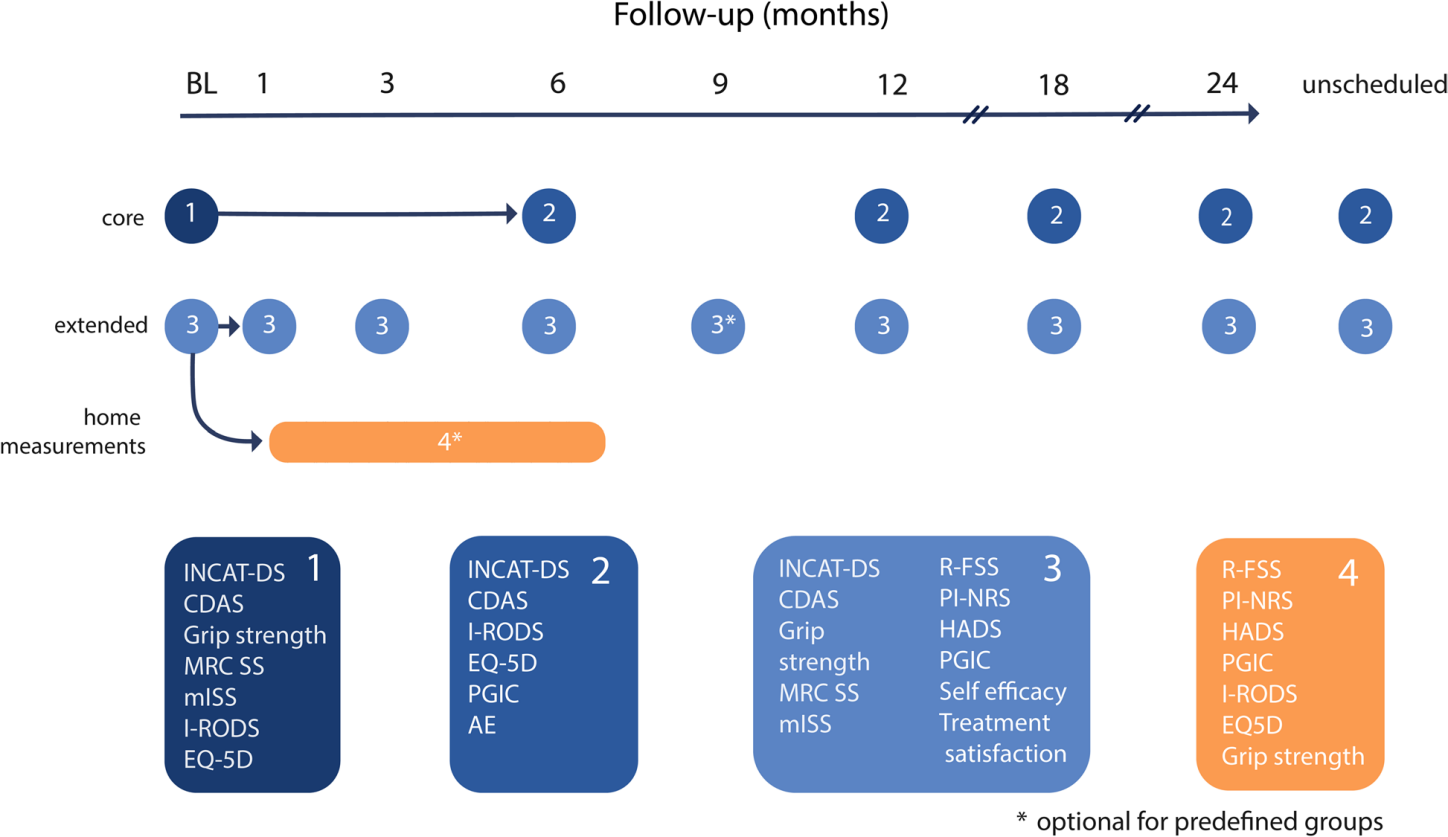
Follow-up schedule and outcome parameters.Visits are planned at minimum every six months (core module), with additional visits for patients starting treatment or treatment withdrawal (extended module). The home-assessment model includes grip strength and patient reported outcomes every two weeks between visits. Additional unscheduled visits can be performed if necessary. INCAT-DS, Inflammatory Neuropathy Cause and Treatment Disability Score. CDAS, CIDP disease activity status. MRC-SS, Medical Research Council Sum Score. mISS, Modified Inflammatory Neuropathy Cause and Treatment Sensory Sum score. I-RODS, Inflammatory Rasch-Overall Disability Scale. EQ-5D, EuroQol quality of life. PGIC, Patient Global Impression of Change. R-FSS, Rasch-built Fatigue Severity Scale. AE, adverse events. PI-NRS, Pain Intensity Numeric Rating Scale. HADS, Hospital Anxiety and Depression Scale

The modular design of the database enables flexibility and ensures a suitable follow-up schedule for every participant. Centers can choose to contribute to all modules or to the core module only, depending on their preference and local logistical support. Follow-up duration for INCbase is a minimum of two years and may be extended for as long as neurological monitoring is indicated. Visits are scheduled at minimum every 6 months (core module), with additional visits at one and three months concurrent with clinical visits for patients with treatment changes (extended module) and home-assessments at scheduled intervals (week 2, 4, 6, 8, 12, and 16) for predefined groups of patients (home-assessment module) (Fig. 1). Unscheduled visits may be conducted to capture deterioration, treatment changes and treatment response. For pediatric cases, a custom follow-up schedule may be determined based on age.

#### Clinical assessment during follow-up

At each new visit, the diagnosis of CIDP is re-confirmed to assess the frequency of change of initial CIDP diagnosis, and determinants thereof. Current treatment, recent change in treatment schedule and adverse events can be captured, if applicable. The minimum follow-up clinical assessments by physicians include the INCAT-DS and grip strength (core module). Additional measures collected include the MRC-SS and mISS, and optionally the 10 m walk test, the 6 min walking test and the timed up and go (TUG) test (extended module). The minimum collected questionnaires (PROMs) in the core module include the Inflammatory Rasch-built Overall Disability Scale (I-RODS) [12], the EuroQol EQ-5D health questionnaire [13] and a 5-point Patient Global Impression of Change (PGIC). The extended and home assessment module include additional questionnaires (R-FSS, PI-NRS, HADS and GSES). For the home-assessments, patients are instructed to measure grip strength at home using a Vigorimeter.

#### Data entry

As a data entry system, a customizable, modular and web-based application was developed. Local investigators enter pseudonimised data directly into the web-based registry. Questionnaires and home measurements are collected by electronic case report forms (eCRF) sent to patient’s email address. Local investigators can select and export fields of choice from each visit as a PDF file that can be used for the patient healthcare electronic record to avoid double entry of data.

#### Biomaterials

Participating centers within INCbase have the option to collect and store biomaterials. Sampling is performed at predefined time points, and processed and stored as serum, DNA, RNA, plasma and peripheral blood mononuclear cells (PBMC) in a local biobank. Selected centers will collect RNA, plasma, PBMCs and serum longitudinally during periods of presumed disease activity (e.g. before treatment initiation and at relapse) and in periods of stable disease (treatment response or remission). If residual materials from routine diagnostic work-up, such as cerebrospinal fluid or skin and nerve biopsy samples, are available, these can be collected and stored. Apart from blood draws, no diagnostic procedures will be repeated for the sole objective to collect biomaterials. Children less than 16 years of age will not have blood sampling for the sole purpose of this study, but if blood is drawn for a separate clinical indication, material may be stored for the study. Contribution of samples to specific collaborative studies is based on an opt-in principle, alternatively, centers can use biomaterials for studies that are within the scope of the INCbase protocol.

#### INCbase governance and INCbase data registry and biomaterial policy

**Fig. 2:**
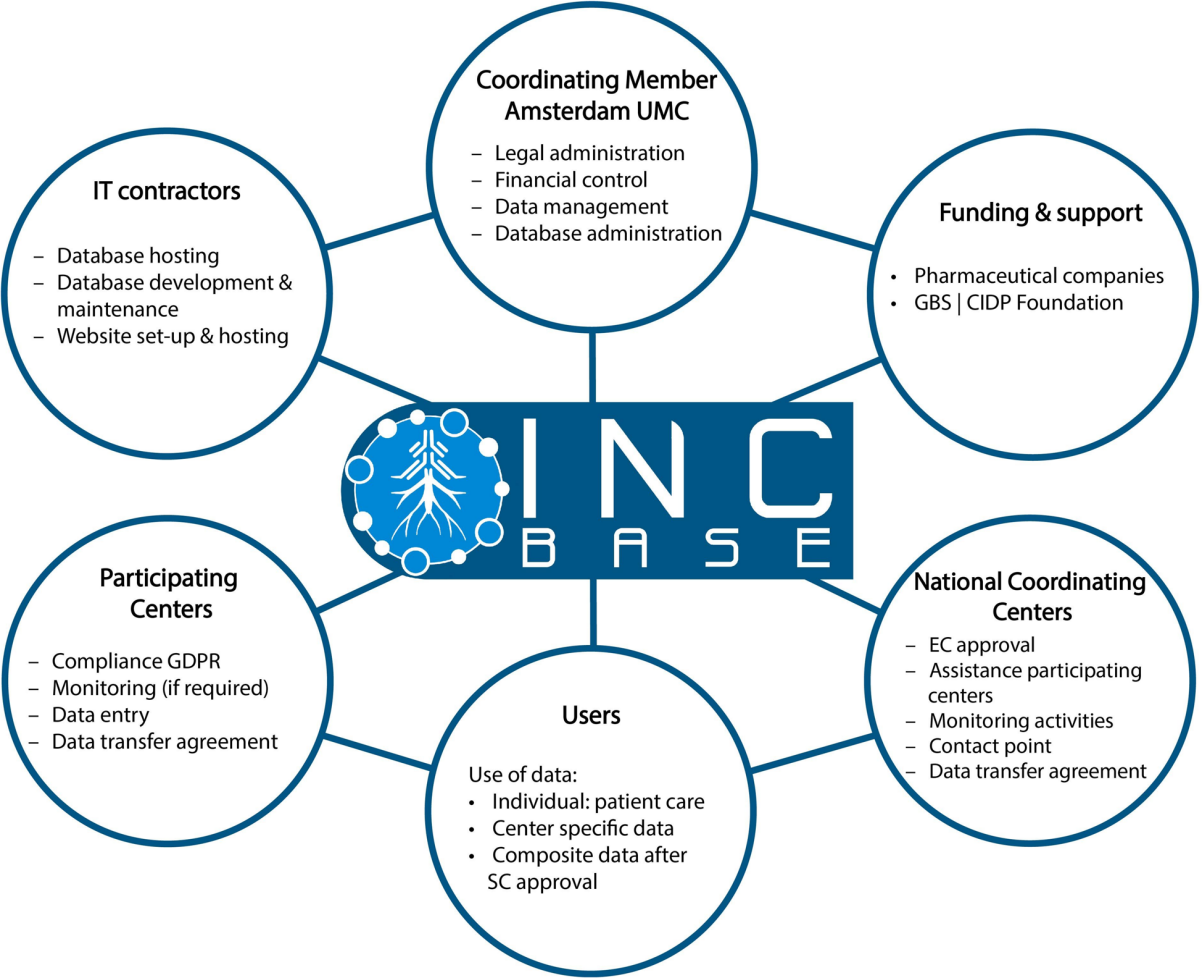
INCbase infrastructure

INCbase governance is provided by the Steering Committee (SC), the Operational Management Team (OMT), a Scientific Advisory Board and a stakeholders and advisory body (Inflammatory neuropathy Consortium Board or INC-board). The Amsterdam UMC acts as Coordinating Member. National Coordinating Centers are responsible for coordinating the INCbase Registry activities in each specific country (Supplementary Table1, Fig. 2). To be eligible to join INCbase, participating members are required to accede to the INCbase Data Registry and Biomaterial Policy. This document defines agreements and principles regarding data and biomaterial sharing between participating centers. Each participating center remains owner of the data it has supplied to the registry. Members may request composite data to be made available by submitting a study proposal to the INCbase Steering Committee.

#### Financial infrastructure

INCbase is a collaborative effort between academic centers, patient groups, and the pharmaceutical industry. Financial support was received from the GBS/CIDP Foundation International and from pharmaceutical companies via investigator-initiated grants for sub-studies within INCbase that included and prioritized mutual objectives to increase our knowledge about CIDP. Funding is being used for the core infrastructure (IT and legal infrastructure) and to a limited extend local support of participating centers.

#### Ethics and informed consent procedure

INCbase obtained approval from the Medical Ethics Committee of Amsterdam UMC in March 2020. Participating centers are required to obtain local approval of the Institutional Review Board (IRB)/ethics committee before accession. The study is conducted in accordance with the ethical principles that have their origin in the Declaration of Helsinki, and are consistent with the international council for harmonization (ICH) Good Clinical Practice (GCP) guidelines and local regulatory requirement(s). The informed consent form includes consent questions regarding participation in the home assessment module, collaborative studies with commercial parties, use of biomaterial in genetic studies and storage and sharing of data and material. In pediatric cases, specific informed consent is required from parents and/or children, depending on age.

#### Privacy

Privacy measures and safeguards are in accordance with the Medical Treatment Contract Act and the General Data Protection Regulation (GDPR, EU-Directive 95/46/EC). Data is pseudonimised, and the key to pseudonimisation is kept only at the local site.

## Methods primary objective: design, outcomes and analyses

### Design

The primary objective is to develop and validate a prediction model for treatment response. Clinical data will be gathered at baseline and at six months (core module) as described above. To ensure widespread clinical applicability in an international setting, we will construct a prediction model based on five, or less, predictor variables. Eligible predictor variables are demographics (age, gender), diagnostic likelihood based on the 2021 EAN/PNS criteria, variant, disease duration, disease course before start of treatment, and baseline disability and impairment measures (i.e. the INCAT-DS, MRC sum score, I-RODS, and grip strength). Other variables might be added in the future as our knowledge of the disease expands (for example biomarkers of disease activity or tissue damage). Design and reporting of the results will be done in accordance with the TRIPOD guidelines [17].The study population will be split into development and validation cohort. From the INCbase population, the following participants are included to develop and validate the prediction model.

### Inclusion criteria primary objective:

1. Fulfilling 2021 EAN/PNS diagnostic criteria for CIDP or possible CIDP (nerve conduction studies weakly supportive of demyelination + one supportive criterion) [1];
2. Treatment naive at baseline, with clinically presumed active disease and sufficient severity of disease to warrant start of first line immunomodulatory treatment (i.e. immunoglobulins, corticosteroids or a combination of both);
3. Availability for follow-up for at least six months;

### Exclusion criteria primary objective:

1. The presence of (para)nodal auto-antibodies.

### Outcomes

Treatment response will be defined as improvement by at least the minimal clinically important difference (MCID). As there is no gold standard for determining treatment response in CIDP, multiple definitions will be employed. For our primary analyses, treatment response will be defined as improvement by the MCID on one disability measure (i.e. a decrease in adjusted INCAT-DS ≥ 1 OR an increase on the I-RODS centile scale ≥ 4). For sensitivity analyses, treatment response will be defined as improvement by the MCID on both disability measures combined, and as improvement by the MCID on a combination of one of the disability measures and muscle strength (i.e. increase of MRC sum score (max. 60) of ≥ 4 or increase of grip strength of ≥ 8 kPa).

### Statistical analysis

To develop a prediction model of treatment response we will use univariate and multivariate logistic regression analysis to first identify possible predictors and subsequently identify the optimal combination of predictors. For the prediction model, we will focus on patients in whom the diagnosis has not been changed during the first year of follow-up. In these models, treatment response is the dependent dichotomous variable. Data missing at random will be handled by multiple imputation by chained equations (MICE). First, univariate associations will be explored for all predictors. Next, to identify the optimal combination of predictors, all predictors will be entered into a multivariate and backward selection with bootstrapping will be employed to reduce the number of predictors. Potential effects of variation in treatment regimen will be assessed in subgroup analyses. Model performance in the development dataset will be assessed through discrimination (c-statistic with 95% confidence interval) and calibration (assessed graphically) and will be internally assessed using bootstrapping. After internal validation, regression coefficients may be adjusted for optimism. Final model performance will be assessed in the validation data set comprising patients not used for model development.

### Sample size

We estimate that a population of 1000 newly diagnosed patients is needed to ensure sufficient numbers of patient not responding to treatment, which we estimate to be around 20% [18]. The cohort will be split into a development and validation cohort. To create a clinically applicable and implementable model we estimate to include five predictor variables. To meet the recommended event rate of 1 per 20 non-responders, 500 patients are needed in the development cohort [19]. For external validation a minimum of 100 patients in the smallest outcome group (i.e. CIDP non-responders) is recommended, leading to a validation cohort of also 500 patients. Additionally, this sample sizes provides a safety margin for changes in diagnosis (expected at roughly 10%) and patients lost to follow-up for secondary analyses [20].

### Dissemination of results

The externally validated prediction model will be made available to researchers and clinicians on the INCbase website as a personalized prediction model providing the predicted probability of treatment response to first line treatment for a CIDP patient given the individual values for the predictors.

## Discussion

Leveraging data from INCbase, a large scale observational study on patients with CIDP, we aim to create a model for the prediction of response to first-line immunomodulatory treatment, to support patients and clinicians in decisions on treatment regimens after a CIDP diagnosis. This model will contribute to adequate treatment selection and timely escalation of treatment, reducing the risk of disease progression and irreversible disability in CIDP patients.

There are several possible limitations to our approach. First, we chose to define treatment response based on improvement by the MCID. Treatment response in CIDP remains a poorly defined and heterogeneous concept. Improvement by the minimal clinical important difference on one outcome measure may not reflect an optimal treatment response, or meaningful improvement as perceived by patient or physician. However, as optimal treatment response is a patient-specific concept and therefore difficult to define, it is not an appropriate outcome for this prediction model. Also, the concept of the MCID and optimal treatment response in CIDP is currently being addressed in clinimetric studies. Therefore, definitions of treatment response could change by the time we will have sufficient patients to develop our prediction model. To prevent an overestimation of treatment response, we included a sensitivity analysis combining multiple outcome measures. Second, the trade-off between model complexity and clinical applicability and interpretability poses a challenge. To avoid an overly complex model, we opted to include around five clinically relevant predictor variables often documented in standard care, ensuring the model remains implementable in clinical practice while still incorporating key factors that may predict treatment response. Third, we chose to construct the model for newly diagnosed patients only. Although this limits generalizability, and prediction of treatment response may also be valuable in previously treated patients, any previous treatment and corresponding response or accumulated nerve damage may require a model with a different set of predictors.

**Fig. 3:**
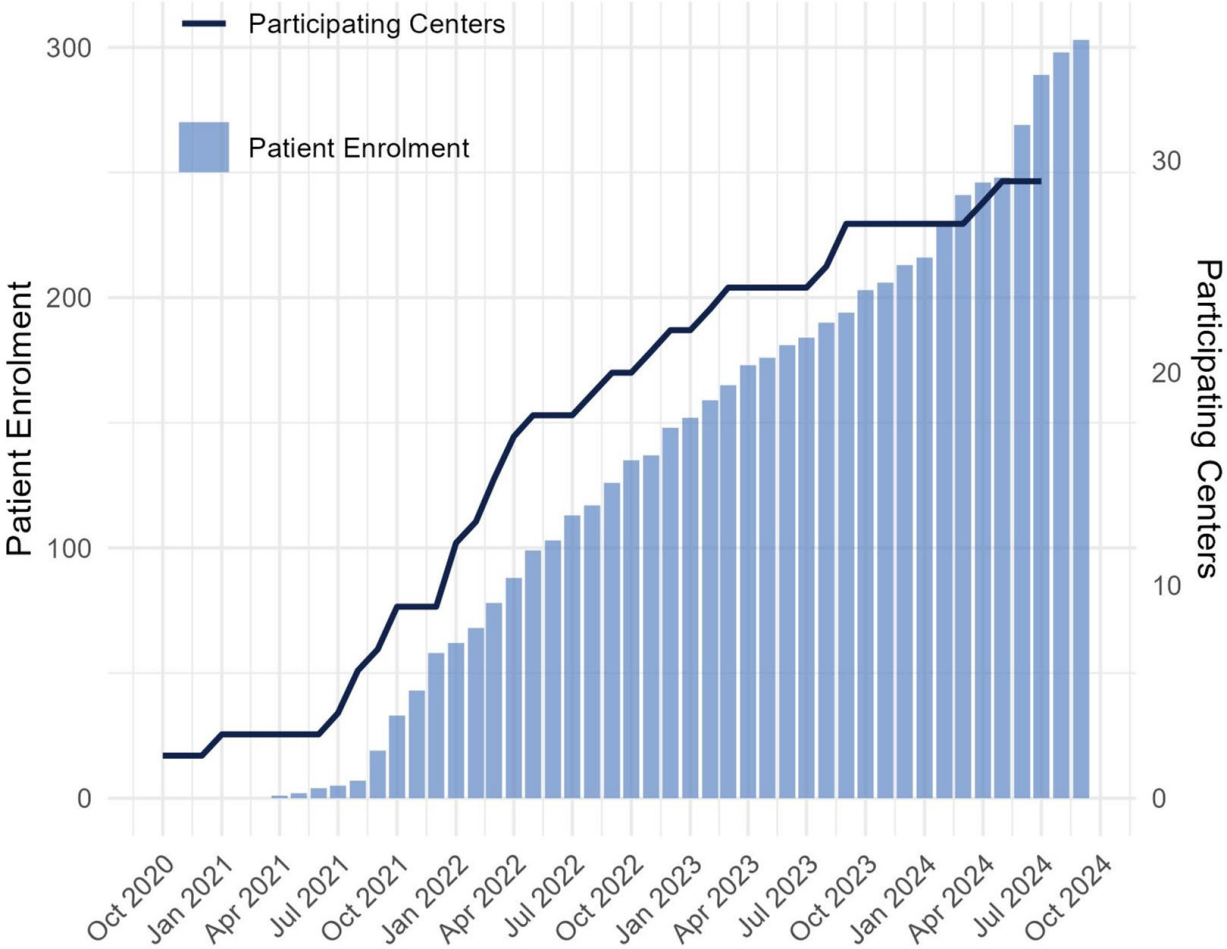
INCbase patient enrolment and center participation

**Fig. 4:**
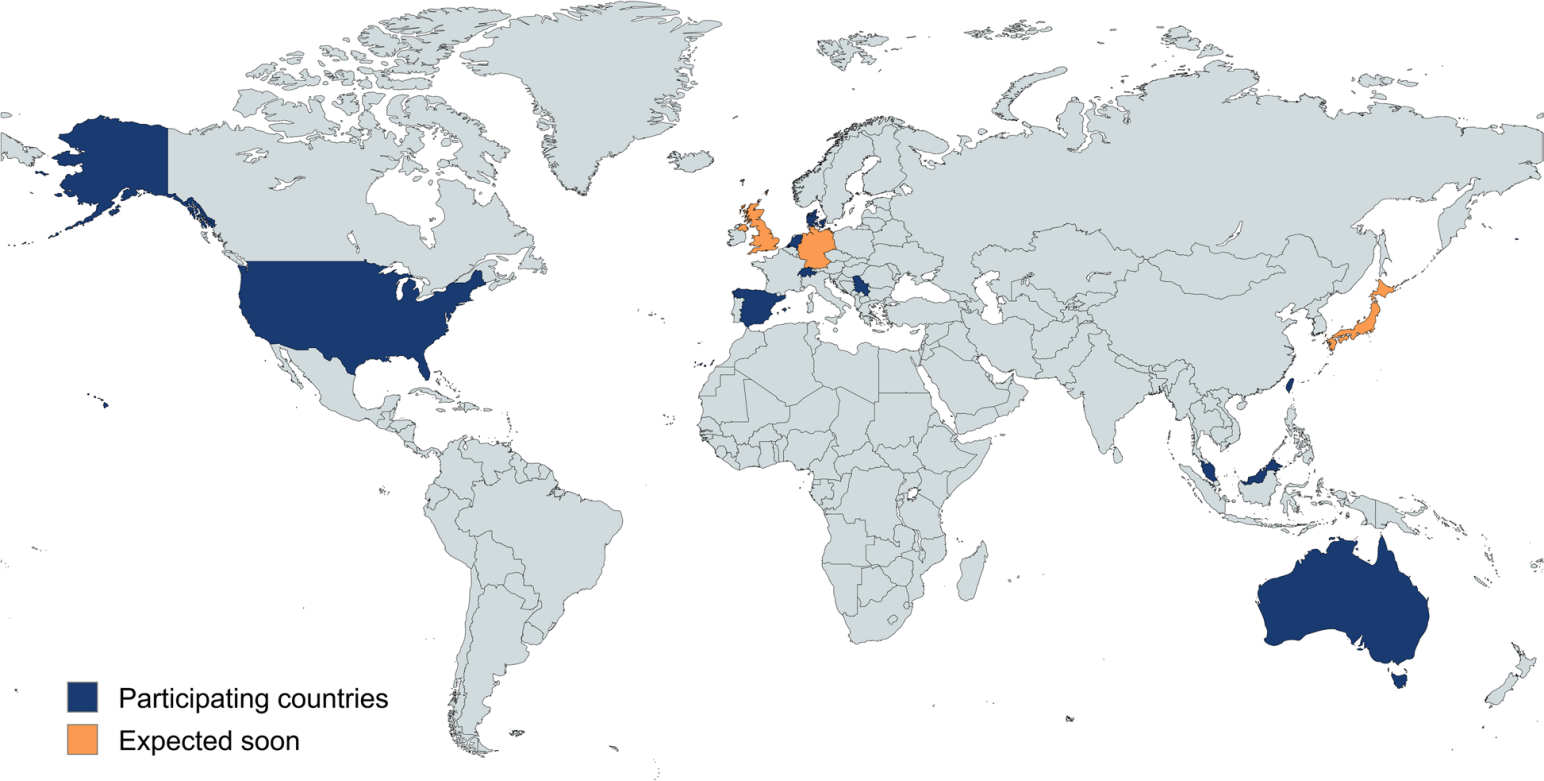
Overview of INCbase participating countries and centers. Image created with mapchart.net

INCbase is an initiative resulting from the 231st European Neuromuscular Center (ENMC) workshop in May 2017, in which the need for standardization of data collection on CIDP and harmonization of registry protocols to enhance future international collaborative research efforts was established [21]. The aim was to create a central registry parallel to ongoing existing registries such as ICOS [8] and the Italian CIDP database, with the future goal to harmonize existing databases with INCbase to ensure global coverage. After consensus was reached on the collection of a minimal core set of clinical and diagnostic data, biomaterials and the infrastructure of the registry, INCbase was created. The first INCbase patient was recruited in Amsterdam UMC in April 2021. Following an initial phase with sparse enrollment due to the COVID pandemic, 29 centers from 8 countries were able to join INCbase and as of September 2024, 303 patients are enrolled (Figs. 3 and 4).

### Future perspectives

As outlined in the introduction, the development of a prediction model for treatment response and improving diagnosis and discovery of biomarkers to guide treatment are our main objectives. In addition, INCbase will also:

1. Characterize the clinical and electrophysiological spectrum of CIDP;
2. Provide understanding how strength impairment, disability, and quality of life impacts patients at various short and long-term stages of their disease;
3. Define the minimal clinical important differences and optimal response when using standard outcome measures;
4. Describe patient and physician satisfaction with different treatments for CIDP.
5. Deepen the knowledge on pathophysiology and underlying immunological pathways.

New modules with additional outcome measures may be developed and incorporated into the database to address future research questions. In addition to these objectives, we aim to expand the geographic footprint of INCbase to centers in less developed countries, which are currently underrepresented in clinical studies in CIDP. We also aim to complete data and biomaterial sharing agreements with parties beyond INCbase, including other CIDP databases (e.g. the Italian and French registries), such that the power of these registries to detect meaningful findings in a rare disease like CIDP can be multiplied. Other disease state registries of interest include IGOS for Guillain-Barre Syndrome [22] and IMAGiNe for paraproteinemic neuropathies [23]. Finally, we are exploring the possibility of using INCbase as a trial infrastructure for phase 2 proof-of-concept studies. Due to the flexible infrastructure and agreement on ownership of data, INCbase is uniquely poised for both large-scale collaborations in CIDP research as well as stimulating smaller, local research in a uniform and reproducible manner. Meanwhile, as INCbase infrastructure is growing, governance and organization of INCbase are further being professionalized. In 2025 we expect to publish various working documents to guide accession for participants centers, introduce a helpdesk for data entry and export, and provide guidance on research projects submissions. More information on INCbase can be found on https://www.incbase.info/.

## Supplementary Information

Supplementary Material 1.

## Data Availability

No datasets were generated or analysed during the current study.

## Abbreviations

AE: Adverse events
CSF: Cerebrospinal fluid
CIDP: Chronic inflammatory demyelinating polyneuropathy
EAN: European Academy of Neurology
ENMC: European Neuromuscular Center
EFNS: European Federation of Neurological Societies
GDPR: General Data Protection Regulation
GSES: General Self Efficacy Scale
HADS: Hospital Anxiety and Depression Scale
ICH-GCP: International council for harmonization- Good Clinical Practice
ICOS: International CIDP Outcome Study
I-RODS: Inflammatory Rasch-built Overall Disability Scale
INC-base: Inflammatory Neuropathy Consortium
INCAT-DS: Inflammatory Neuropathy Cause and Treatment Disability Score
IVIg: Intravenous immunoglobulins
MRC-SS: Medical Research Council sum score
MMN: Multifocal motor neuropathy
mISS: Modified Inflammatory Neuropathy Cause and Treatment Sensory Sum Score
OMT: Operational management team
PBMC: Peripheral blood mononuclear cells
PE: Plasma exchange
PGIC: Patient global impression of change
PI-NRS: Pain Intensity Numerical Rating Scale
PNS: Peripheral Nerve Society
POEMS: Polyneuropathy, Organomegaly, Endocrinopathy, Monoclonal Gammopathy and Skin Changes
PROM: Patient reported outcome measure
Rasch-FSS: Rasch-built-7-item modified fatigue severity scale
SCIg: Subcutaneous immunoglobulins
SC: Steering Committee
