# Supplementary material for: Inflammatory Neuropathy Consortium base (INCbase): a protocol of a global prospective observational cohort study for the development of a prediction model for treatment response in chronic inflammatory demyelinating polyneuropathy
Source: BMC Neurol. 2024 Oct 25;24:415. doi: 10.1186/s12883-024-03903-w (PMC11515301; doi:10.1186/s12883-024-03903-w)
Supplement: Supplementary file 1 — Supplementary Material 1. [file 12883_2024_3903_MOESM1_ESM.docx]

## Additional file 1. to Inflammatory Neuropathy Consortium Base (INCbase): a protocol for a global prospective observational cohort study on chronic inflammatory demyelinating polyneuropathy

**Table 1. Centers currently participating in INCbase (September 2024)**

| **Country** | **Center** | **Role** |
| --- | --- | --- |
| The Netherlands | **Amsterdam University Medical Center, location AMC** | **Coordinating member** |
| Australia | **Alfred Hospital, Melbourne** | **National coordinating center** |
| Denmark | **Aarhus University Hospital, Aarhus** | **National coordinating center** |
| Malaysia | **University of Malaya, Kuala Lumpur** | **National coordinating center** |
| Serbia | **University Clinical Centre of Serbia, Belgrade** | **National coordinating center** |
| Spain | **Hospital de la Santa Creu i Sant Pau** | **National coordinating center** |
|  | Hospital Universitario Infanta Sofia  Hospital Universitario de Navarra  Hospital Universitario y Politècnico la Fe  Hospital Universitario Central de Asturias  Fundació Clinic per la Recerca Biomèdica  Hospital Unversitari Vall d’hebron | Participating centers |
| Switzerland | **Neurocenter of Southern Switzerland** | **National coordinating center** |
|  | Lausanne University Hospital  HFR Fribourg Cantonal Hospital  Cantonal Hospital of St. Gallen  University Hospital of Basel  University Hospital of Geneva | Participating Centers |
| Taiwan | **National Taiwan University Hospital** | **National coordinating center** |
|  | Taipei Municipal Wan Fang Hospital  Chang Gung Memorial Hospital, Linkou Medical Center  Taipei Veterans General Hospital | Participating Centers |
| USA | **University of Minnesota Medical Center** | **National coordinating center with the right to subcontract**  **participating centers** |
|  | Johns Hopkins University Hospital  Kansas University Medical Center  University of Michigan Hospital  Duke University Hospital  Lahey Clinic  NeuroMD Center | Participating Centers |
